# ST6Gal-I overexpression facilitates prostate cancer progression via the PI3K/Akt/GSK-3β/β-catenin signaling pathway

**DOI:** 10.18632/oncotarget.11699

**Published:** 2016-08-30

**Authors:** Anwen Wei, Bo Fan, Yujie Zhao, Han Zhang, Liping Wang, Xiao Yu, Qingmin Yuan, Deyong Yang, Shujing Wang

**Affiliations:** ^1^ Department of Biochemistry and Molecular Biology, Institute of Glycobiology, Dalian Medical University, Dalian, 116044, Liaoning Province, China; ^2^ Department of Urology, First Affiliated Hospital of Dalian Medical University, Dalian, 116011, Liaoning Province, China; ^3^ Department of Pathology, Dalian Medical University, Dalian, 116044, Liaoning Province, China

**Keywords:** ST6Gal-I, α2,6-sialic acid, prostate cancer, prognosis, proliferation and invasion

## Abstract

ST6Gal-I sialyltransferase adds α2,6-linked sialic acids to the terminal ends of glycan chains of glycoproteins and glycolipids. ST6Gal-I is reportedly upregulated in many cancers, including hepatocellular carcinoma, ovarian cancer and breast cancer. However, the expression and function of ST6Gal-I in prostate cancer (PCa) and the mechanism underlying this function remain largely unknown. In this study, we observed that ST6Gal-I expression was upregulated in human PCa tissues compared to non-malignant prostate tissues. High ST6Gal-I expression was positively correlated with Gleason scores, seminal vesicle involvement and poor survival in patients with PCa. ST6Gal-I knockdown in aggressive prostate cancer PC-3 and DU145 cells significantly inhibited the proliferation, growth, migration and invasion capabilities of these cells. ST6Gal-I knockdown decreased the levels of several PI3K/Akt/GSK-3β/ β-catenin pathway components, such as p-PI3K, (Ser473)p-Akt, (Ser9)p-GSK-3β and β-catenin. Furthermore, targeting this pathway with a PI3K inhibitor or Akt RNA interference decreased p-Akt, p-GSK-3β and β-catenin expression, resulting in decreased PC-3 and DU145 proliferation, migration and invasion. Taken together, these results indicate that ST6Gal-I plays a critical role in cell proliferation and invasion via the PI3K/Akt/GSK-3β/β-catenin signaling pathway during PCa progression and that it might be a promising target for PCa prognosis determination and therapy.

## INTRODUCTION

PCa is a growing public health problem worldwide and is the most common malignant neoplasm diagnosed in men in the United States and Western Europe, as well as the leading cause of death among men [1]. The incidence of PCa has risen each year, and more than 30,000 men are diagnosed annually in China [2]. During its initial stages, PCa is not only responsive to androgen ablation therapy but also characterized by slow growth and thus usually remains confined to the prostate gland itself and causes only limited symptoms. As PCa advances, it often becomes androgen-refractory and spreads beyond the prostate and invades surrounding tissues. Eventually, a metastatic PCa phenotype develops in affected patients [3]. Therefore, elucidating the molecular mechanism underlying PCa development and progression may be helpful in detecting the disease, as well as in determining patient prognosis and individualizing therapy.

Sialic acids are widely expressed as terminal glycans on glycoconjugates of eukaryotic cells. Sialylation is associated with a variety of cellular functions, such as cell adhesion, signal recognition and protein stability [4–6]. More than twenty distinct sialyltransferases have been identified in the human genome, and these enzymes catalyse the transfer of sialic acid from CMP-Neu5Ac to the terminal glycans of glycoconjugates. Based on differences in glycosidic linkages and monosaccharide acceptors, sialyltransferases are divided into four protein families (ST3Gal, ST6Gal, ST6GalNAc and ST8Sia) [7]. Increasing evidence indicates that abnormal sialylation is closely related to malignant tumour phenotypes, including proliferative, invasive, metastatic and drug-resistant phenotypes [8–11].

ST6Gal-I is the main sialyltransferase responsible for α2,6-linked sialic acid formation on N-glycans. ST6Gal-I is reportedly highly expressed in many cancers, including hepatocellular carcinoma, colon cancer, renal carcinoma and ovarian cancer [11–15]. For example, Liu et al. demonstrated that high ST6Gal-I expression in localized clear-cell renal cell carcinoma was predictive of worse postoperative survival and higher recurrence rates [15]. Ferreira et al. reported that high ST6Gal-I expression is associated with greater skin tumour invasiveness and metastatic potential [16]. Moreover, our previous study showed that ST6Gal-I upregulation can increase the adhesion of mouse hepatocarcinoma Hca-F cells to lymph nodes [17]. These results indicate that ST6Gal-I overexpression not only promotes malignant phenotypes of tumor cells but also predicts patient prognoses. It has also been reported that ST6Gal-I overexpression facilitates cisplatin resistance in ovarian cancer cells [10] and that ST6Gal-I knockdown reverses human leukaemia multidrug resistance by downregulating P-glycoprotein and multidrug resistance related protein 1 expression [18]. Meanwhile, radiotherapy induces α2,6-sialylation upregulation on β1 integrins and the EGFR in cancer cells, which increases cellular adhesion and migration ability [14, 19]. These findings suggest that ST6Gal-I regulates the effects of radiotherapy and chemotherapy. However, the role of ST6Gal-I in PCa and the molecular mechanisms by which it mediates cell migration and invasion remain poorly understood.

In this study, we reported the clinical relevance of ST6Gal-I in PCa and showed that ST6Gal-I is required to maintain PCa cell aggressiveness and malignant properties. ST6Gal-I expression increased with PCa progression and was closely correlated with clinical survival and seminal vesicle invasion. ST6Gal-I knockdown effectively inhibited PCa cell proliferation, migration and invasion ability. Furthermore, the expression levels of p-Akt, p-GSK-3β and β-catenin in the PI3K/Akt/GSK-3β/β-catenin signaling pathway were decreased upon ST6Gal-I downregulation. Wortmannin, a PI3K/Akt/GSK-3β/β-catenin pathway inhibitor, and Akt RNA interference significantly inhibited the proliferation, migration and invasion ability of PCa cells. Thus, ST6Gal-I may be a promising target for early PCa diagnosis and treatment.

## RESULTS

### High ST6Gal-I expression is positively correlated with Gleason scores and seminal vesicle involvement in PCa patients

**Figure 1:**
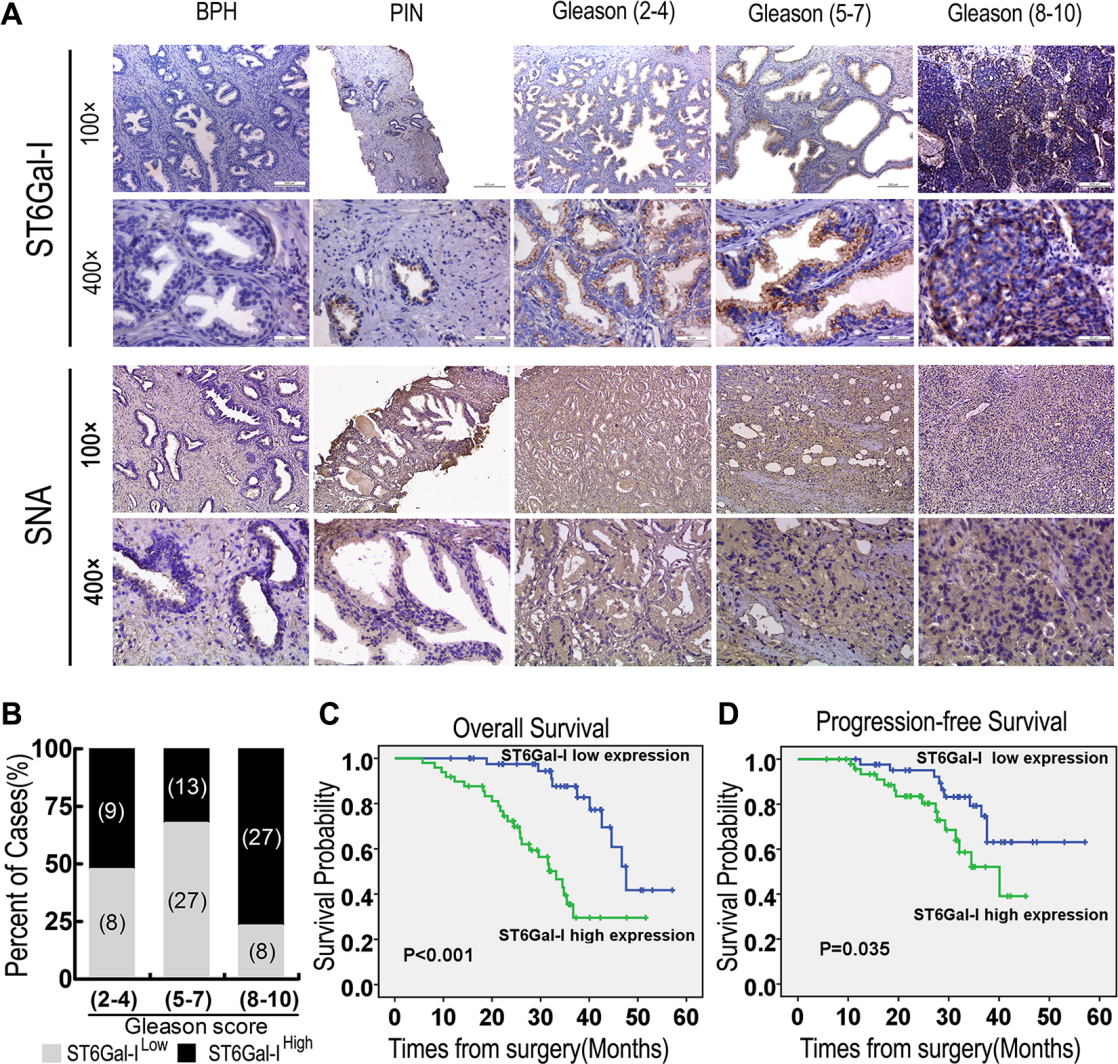
ST6Gal-I upregulation in PCa tissues was correlated with patient prognosis (**A**) Benign prostatic hyperplasia (BPH), prostatic intraepithelial neoplasia (PIN) and PCa tissue samples with low, moderate and high Gleason scores exhibited dynamic increases in ST6Gal-I expression. Representative images of ST6Gal-I IHC staining are shown. (**B**) Specific cases with different Gleason scores exhibiting low and high ST6Gal-I expression are shown. (**C** and **D**) Higher ST6Gal-I expression was associated with the poorer overall survival and poorer progression-free survival. SNA, *Sambucus nigra* agglutinin.

To investigate the clinical relevance of ST6Gal-I expression in PCa, ST6Gal-I and α2,6-linked sialic acid levels in patient tumour lesions were assessed by immunohistochemistry. As shown in Figure 1A, higher ST6Gal-I and α2,6-linked sialic acid expression levels were observed in PCa tissues than in benign prostatic hyperplasia and prostatic intraepithelial neoplasia tissues (*P* < 0.05). According to staining intensity, we subdivided 92 PCa patients into the following two groups: a “low-ST6Gal-I” group containing 43 samples characterized by low ST6Gal-I staining (negatively and weakly stained) and a “high-ST6Gal-I” group containing 49 samples characterized by high staining (moderately and strongly stained, Table 1). The associations between ST6Gal-I expression and PCa clinicopathologic characteristics, including patient age, pretreatment PSA level, Gleason score, pathologic stage, lymph node metastasis, seminal vesicle involvement and perineural invasion, are summarized in Table 1. Gleason scores were found to be positively correlated with ST6Gal-I expression (*P* = 0.001). Specific cases with high and low ST6Gal-I expression in different Gleason score groups were shown in Figure 1B. Spearman's correlation analysis yielded a correlation coefficient of 0.279 (*P* = 0.007) for ST6Gal-I expression and Gleason scores. Moreover, seminal vesicle involvement was also found to be positively correlated with ST6Gal-I expression (*P* = 0.007), and the correlation coefficient for ST6Gal-I expression and seminal vesicle involvement was 0.280 (*P* = 0.007). These results suggest that ST6Gal-I overexpression is significantly associated with PCa progression.

**Table 1:** Distribution of characteristics of patients with PCa by ST6Gal-I expression

Patient characteristics	ST6Gal-I expression		Total	*P* Value
	Low	High		
Age, years				0.856
< 71	21	23	44	
≥ 71	22	26	48	
Pretreatment PSA				0.160
< 10	29	26	55	
≥ 10	14	23	37	
Gleason score				**0.001**
2–4	8	9	17	
5–7	27	13	40	
8–10	8	27	35	
Pathologic stage				0.062
T2a/T2b	22	16	38	
T3a/T3b	18	22	40	
T4	3	11	14	
Lymph node metastases				0.204
Negative	37	37	74	
Positive	6	12	18	
Seminal vesicle involvement				**0.007**
Negative	35	27	62	
Positive	8	22	30	
Perineural invasion				0.526
Negative	29	36	65	
Positive	14	13	27	

Abbreviation: PSA, prostate specific antigen.

### High ST6Gal-I expression is related to poor overall survival and poor progression-free survival in PCa patients

To determine whether ST6Gal-I expression level is a significant predictor of overall survival after radical prostatectomy, Kaplan-Meier curves were calculated to determine the relationships between high or low ST6Gal-I expression and overall survival. The overall survival of patients with high ST6Gal-I expression was significantly lower than that of patients with low ST6Gal-I expression (*P* < 0.001; Figure 1C). On univariate analysis, high pretreatment PSA levels, higher Gleason scores, higher pathologic stages, lymph node metastases, seminal vesicle involvement and high ST6Gal-I expression levels were correlated with poor overall survival in PCa patients. Multivariate analysis showed that high ST6Gal-I expression (HR = 3.603; 95% CI = 1.548–8.387; *P* = 0.003) and higher pathologic stage (HR = 2.119; 95% CI = 1.063–4.226; *P* = 0.033) were independent prognostic factors for overall survival (Supplementary Table S1). Progression-free survival was significantly shorter in high-ST6Gal-I expression patients than in low-ST6Gal-I expression patients (*P* = 0.035; Figure 1D). These associations were confirmed by Cox univariate analysis (HR = 2.873; 95% CI = 1.305–6.325; *P* = 0.009) and multivariate analysis following adjustments for pretreatment PSA, Gleason score and pathologic stage (HR = 2.389; 95% CI = 1.030–5.542; *P* = 0.042) (Supplementary Table S2). These results indicate that ST6Gal-I up-regulation is associated with poor PCa patient prognosis.

### ST6Gal-I downregulation decreases α2,6-linked sialic acid levels in PC-3 and DU145 cells

**Figure 2:**
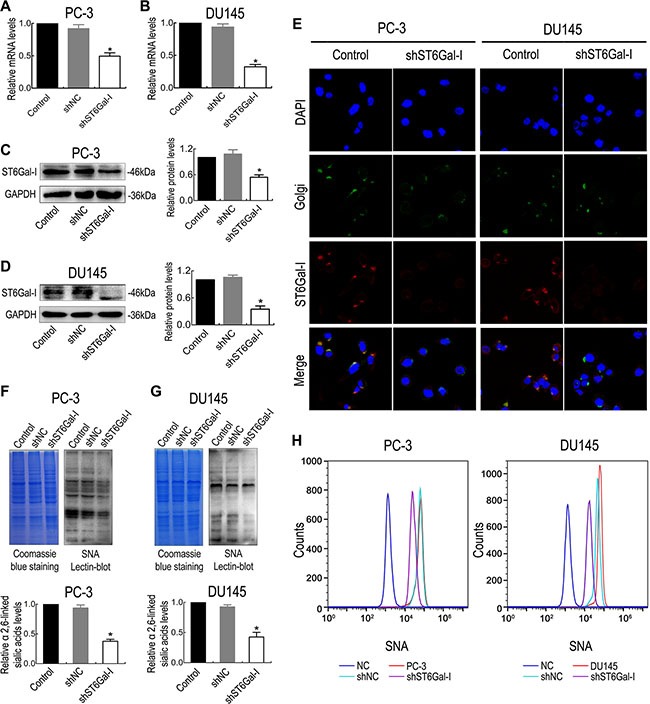
The shRNA interference vector effectively silenced ST6Gal-I expression in PC-3 and DU145 cells The mRNA and protein levels of ST6Gal-I in PC-3 and DU145 cells were significantly reduced after *in vitro* shRNA transfection, as measured by real-time PCR (**A** and **B**), Western blot assay (**C** and **D**) and immunofluorescence (**E**). α2,6-linked sialic acid expression levels in PC-3 and DU145 cells were decreased after ST6Gal-I knockdown, as determined by lectin blotting (**F** and **G**) and flow cytometry assay (**H**). Coomassie Brilliant Blue staining (CBBS) was used as a loading control. Values are from three independent experiments. All values are the mean ± SD of three independent experiments. * represents *P* < 0.05.

To determine if ST6Gal-I is biologically significant with respect to PCa cell aggressiveness, PC-3 and DU145 cells, which express high levels of ST6Gal-I, were subjected to ST6Gal-I knockdown. Two stably silenced ST6Gal-I cell lines were established, and RT-PCR results showed that ST6Gal-I-shRNA transfection inhibited ST6Gal-I mRNA expression compared to untransfected cells and cells transfected with negative control shRNA (Figure 2A and 2B). ST6Gal-I protein levels were also significantly suppressed in ST6Gal-I-shRNA transfected cells compared with negative control cells (Figure 2C and 2D), and this result was confirmed by immunofluorescence (Figure 2E). In addition, lectin blot assay showed that ST6Gal-I knockdown significantly inhibited α2,6-linked sialic acid expression, as shown by *Sambucus nigra* agglutinin (SNA) staining (Figure 2F and 2G). To further confirm the changes in cell surface α2,6-linked sialic acid levels, we performed flow cytometry assay, the results of which showed that cell surface α2,6-linked sialic acid levels were significantly reduced after ST6Gal-I knockdown (Figure 2H). Thus, ST6Gal-I-shRNA effectively reduces ST6Gal-I expression and decreases α2,6-linked sialic acid levels in PC-3 and DU145 cells.

### ST6Gal-I silencing suppresses PC-3 and DU145 cell proliferation and colony formation ability

**Figure 3:**
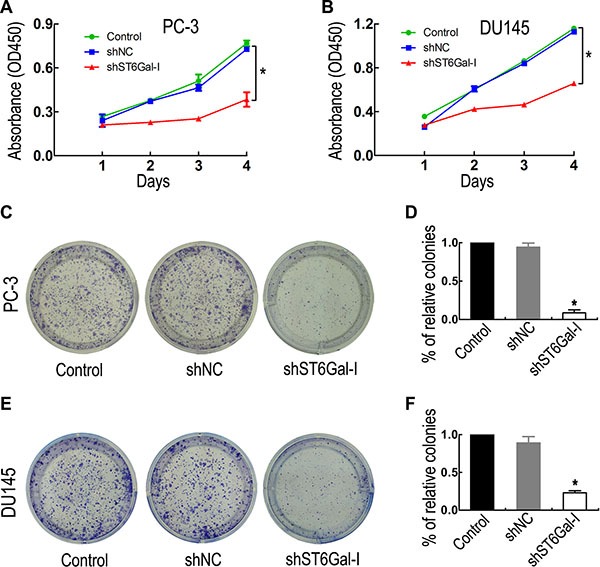
ST6Gal-I downregulation inhibited PCa cell proliferation and colony formation ability Cell proliferation was significantly reduced in the ST6Gal-I-downregulation group compared to the control and shNC groups, as measured by CCK8 assay (**A** and **B**) and colony formation assay (**C** and **E**). (**D** and **F**) The relative cell colony formation rates of PC-3 and DU145 cells from three independent experiments. The CCK8 assay was performed in triplicate. *means *P* < 0.05. The *P* values of the CCK8 assay were calculated by one-way ANOVA.

To investigate the effects of ST6Gal-I knockdown on PCa cell proliferation and colony formation ability, CCK8 and colony formation assays were performed. CCK8 assay showed that PC-3 and DU145 cell proliferation ability was significantly inhibited in the shST6Gal-I group compared with the control and shNC groups (Figure 3A and 3B). To confirm this result, colony formation assay was used to examine cell proliferation changes. Consistently with the CCK8 assay results, ST6Gal-I-I knockdown decreased PC-3 (Figure 3C and 3D) and DU145 (Figure 3E and 3F) cell colony formation. Thus, ST6Gal-I knockdown significantly attenuates PC-3 and DU145 cell proliferation and colony formation ability.

### ST6Gal-I silencing inhibits PC-3 and DU145 cell migration and invasion

**Figure 4:**
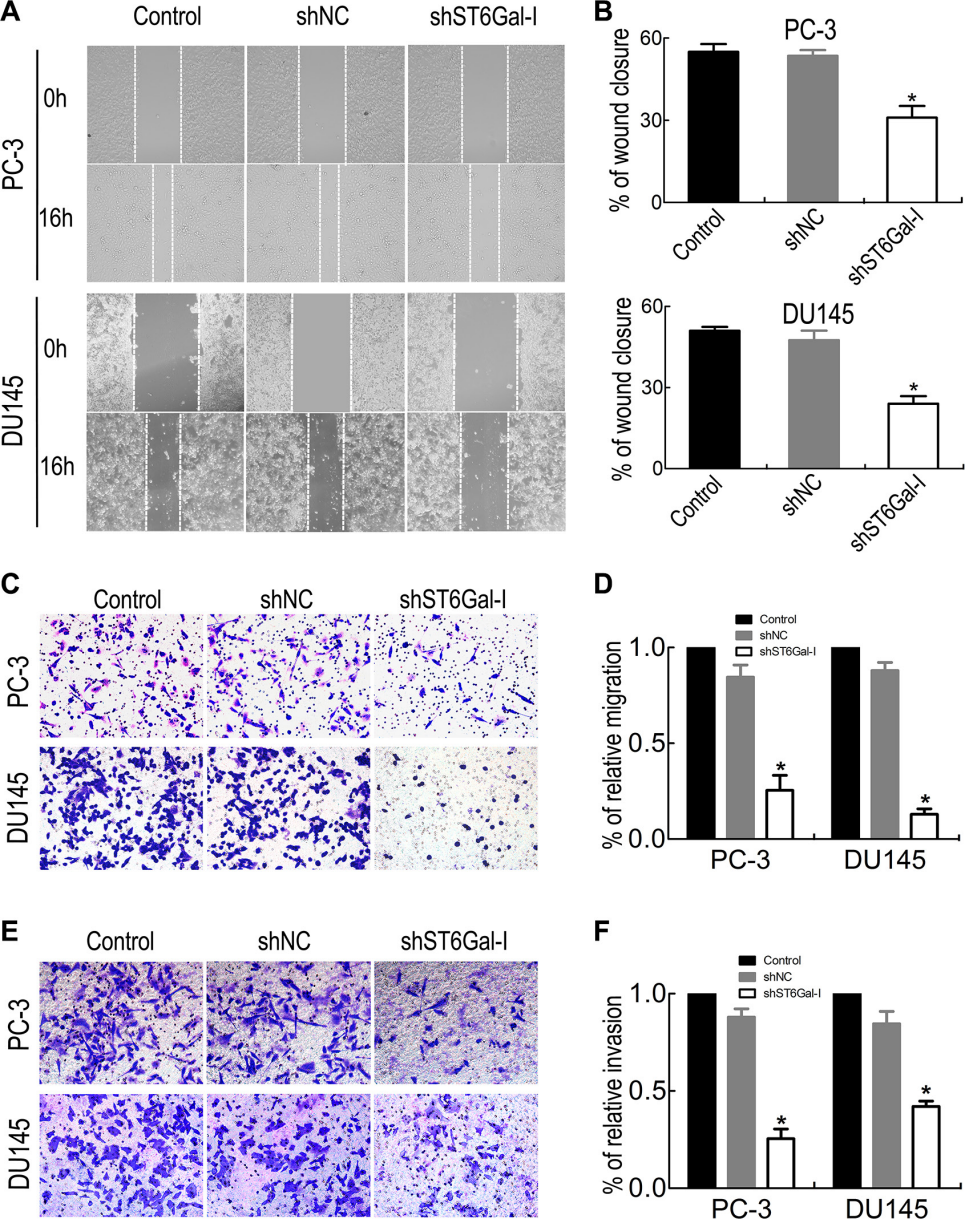
ST6Gal-I knockdown suppressed PCa cell migration and invasion PC-3 and DU145 migration and invasion ability were significantly decreased after ST6Gal-I shRNA transfection, as shown by wound healing assay (**A** and **B**), migration assay (**C** and **D**), and Matrigel invasion assay (**E** and **F**). Values are the mean ± SD of three independent experiments (*P < 0.05).

Cancer metastasis is associated with the poor survival in PCa patients; therefore, we measured the effects of ST6Gal-I silencing on PCa cell migration and invasion. We used wound healing assay to compare changes in migration ability before and after shST6Gal-I vector transfection in PC-3 and DU145 cells. The results showed that ST6Gal-I downregulation significantly inhibited PC-3 and DU145 cell migration (Figure 4A and 4B). Transwell migration assay was also used to analyze PCa cell migration ability, and the results were consistent with those of the wound healing assay (Figure 4C and 4D). Furthermore, Matrigel invasion assay showed that ST6Gal-I knockdown decreased PCa cell invasion (Figure 4E and 4F). These results indicate that ST6Gal-I downregulation attenuates PCa cell migration and invasion ability.

### ST6Gal-I knockdown inhibits the PI3K/Akt/GSK-3β/β-catenin signaling pathway in PC-3 and DU145 cells

**Figure 5:**
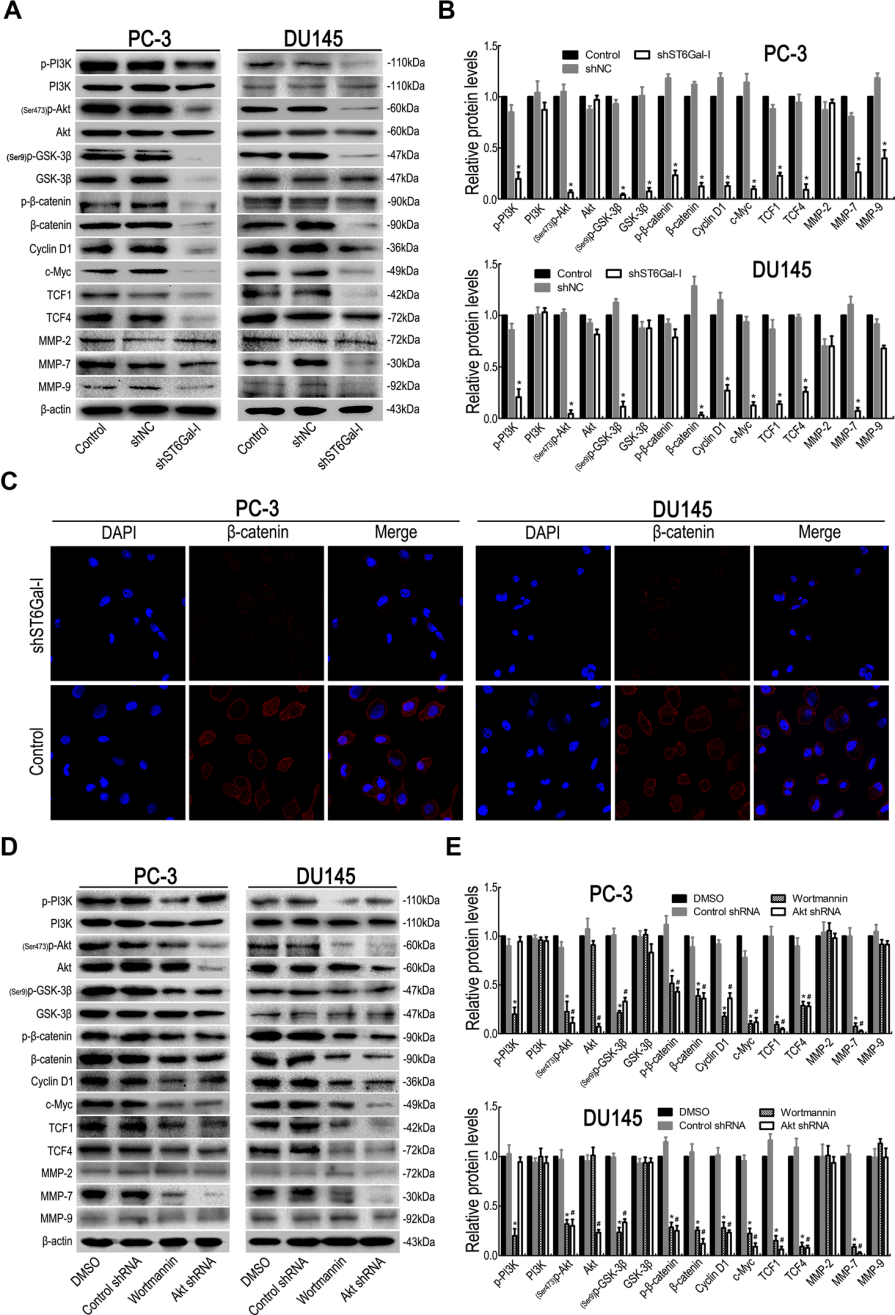
Silencing ST6Gal-I inhibited PI3K/Akt/GSK-3β/β-catenin signaling in PC-3 and DU145 cells The main protein components of the PI3K/Akt/GSK-3β/β-catenin signaling pathway in PC-3 and DU145 cells were measured by Western blot assay (**A**). (**B**) Relative protein intensities were detected by Image Lab software (Bio-Rad). (**C**) The expression levels of nuclear and cytoplasmic β-catenin in PC-3 and DU145 cells upon ST6Gal-I downregulation, as determined via immunofluorescence assay. (**D** and **E**) PCa cells were pretreated with wortmannin and Akt RNAi, and PI3K/Akt/GSK-3β/β-catenin pathway signaling molecule expression levels were analyzed by Western blotting. *P < 0.05 versus DMSO treatment group; ^#^*P* < 0.05 versus control shRNA treatment group.

To elucidate the underlying mechanism by which ST6Gal-I knockdown suppresses PCa cell proliferation, migration and invasion, we analyzed the molecular expression of the PI3K/Akt/GSK-3β/β-catenin signaling pathway and related metastasis regulators in ST6Gal-I-knockdown and control PCa cells. The results showed that ST6Gal-I knockdown decreased the protein levels of p-PI3K, p-Akt, p-GSK-3β and total β-catenin in PC-3 and DU145 cells and inhibited the expression of downstream molecules, such as c-Myc, TCF1, TCF4 and MMP-7 (Figure 5A and 5B). In addition, we noted greater PI3K/Akt signaling activity in PC-3 cells than in DU145 cells. To further determine nuclear and cytoplasmic β-catenin levels in PC-3 and DU145 cells upon ST6Gal-I knockdown, we performed immunofluorescence assay. The results showed that ST6Gal-I downregulation decreased both nuclear and cytoplasmic β-catenin expression (Figure 5C). Taken together, these data indicate that the PI3K/Akt/GSK-3β/β-catenin pathway is associated with reductions in PCa cell proliferation, migration and invasion capability facilitated by ST6Gal-I knockdown.

### Blocking PI3K/Akt/GSK-3β/β-catenin signaling suppresses PC-3 and DU145 cell proliferation, migration and invasion

**Figure 6:**
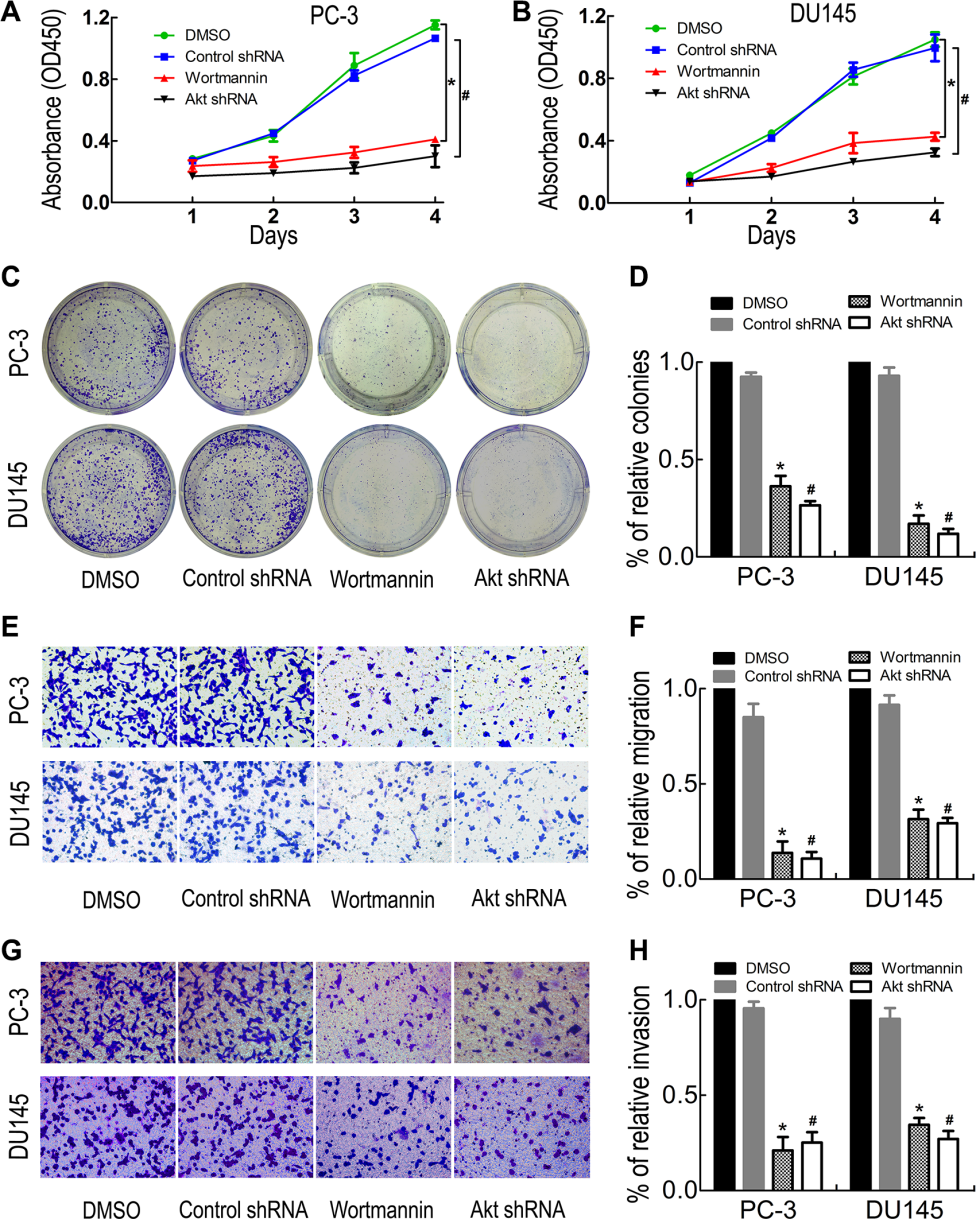
PI3K inhibition reduced PC-3 and DU145 cell proliferation, migration and invasion Wortmannin and Akt shRNA treatment significantly decreased PC-3 and DU145 cell proliferation, migration and invasion ability, as shown by CCK8 assay (**A** and **B**), colony formation assay (**C** and **D**), transwell migration assay (**E** and **F**) and invasion assay (**G** and **H**). *P < 0.05 versus DMSO treatment group; ^#^*P* < 0.05 versus control shRNA treatment group.

To further determine the role of the PI3K/Akt/GSK-3β/β-catenin signaling pathway in PC-3 and DU145 cell proliferation, migration and invasion, PC-3 and DU145 cells were treated with the PI3K inhibitor wortmannin and Akt interference. The results showed that wortmannin and Akt shRNA treatment significantly reduced (Ser473)p-Akt, (Ser9)p-GSK-3β, β-catenin, c-Myc, Cyclin D1, TCF1, TCF4 and MMP-7 expression (Figure 5D and 5E). Meanwhile, the effects of PI3K/Akt/GSK-3β/β-catenin signaling inhibition on PC-3 and DU145 cell proliferation, migration and invasion ability were evaluated by CCK- 8 assay (Figure 6A and 6B), colony formation assay (Figure 6C and 6D), migration assay (Figure 6E and 6F) and invasion assay (Figure 6G and 6H). The results showed that PI3K/Akt pathway inhibition suppressed PC-3 and DU145 cell proliferation, migration and invasion ability. Taken together, these results confirmed that ST6Gal-I silencing inhibits PC-3 and DU145 cell proliferation, migration and invasion ability via the PI3K/Akt/GSK-3β/β-catenin signaling pathway.

## DISCUSSION

In this study, we reported that ST6Gal-I expression was significantly upregulated in PCa tumour tissues and that elevated ST6Gal-I expression was correlated with Gleason scores and PCa prognosis. Based on these findings, we believe that ST6Gal-I plays a key role in promoting malignant tumour progression. Here, we proved that ST6Gal-I silencing significantly attenuates PCa cell proliferation, migration and invasion capability. As mentioned previously, ST6Gal-I overexpression has been observed in many cancers, indicating that ST6Gal-I upregulation might be a common tumour characteristic. Thus, treatments targeting ST6Gal-I may have promise as anti-tumour therapies.

Glycogen synthase kinase-3β (GSK-3β) is widely known for its essential role in triggering β-catenin destabilization in the Wnt/β-catenin signaling pathway, which is hyper-activated in PCa [20]. In PI3K/Akt/GSK-3β/β-catenin signaling pathway, GSK-3β plays an important role in β-catenin phosphorylation and degradation and thus prevents β-catenin from translocating into the nucleus, in which it forms a complex with TCF transcription factor family members to enhance β-catenin-responsive c-Myc, Cyclin D1 and MMP-7 gene expression. Furthermore, GSK-3β activity is inhibited by Akt phosphorylation at Ser9. It has been reported that Akt/GSK-3β signaling suppression reduces β-catenin levels, thereby abrogating gastric cancer cell invasiveness [21]. Consistent with these findings, we found that ST6Gal-I knockdown inhibited the PI3K/Akt/GSK-3β/β-catenin signaling pathway, resulting in the suppression of malignant PCa cell phenotypes. However, GSK-3β itself plays different roles in different cases. Previous studies have shown that GSK-3β overexpression plays key roles in multiple types of cancer, including ovarian cancer [22], breast cancer [23], pancreatic cancer [24–26], colon cancer [27], bladder cancer [28], myeloma [29] and leukaemia [30]. Sun et al. reported that GSK-3β controls PCa cell autophagy and that GSK-3β inhibition triggers autophagy [31]. In addition, it has been reported that GSK-3 suppression reduces PCa cell growth *in vivo* by facilitating the accumulation of C/EBPα protein, a suppressor of E2F1 transactivation [32]. Moreover, Schutz et al. also reported that GSK-3β inhibition decreases PCa cell proliferation *in vivo* by facilitating rapid nuclear exportation of androgen receptor [33]. Furthermore, Kroon et al. demonstrated that GSK-3β plays a pivotal role in maintaining PCa stem cells and that GSK-3β inhibition results in loss of tumourigenicity and attenuated metastatic growth via disrupting F-actin polymerization, which is independent of Wnt signaling [34]. All these results indicate that GSK-3β plays multiple roles in PCa beyond β-catenin phosphorylation and degradation. The underlying mechanisms of these roles need to be further studied.

Ectopic cell surface receptor N-glycosylation resulting from oncogenic transformation has been extensively linked to tumourigenicity and tumour progression regulation. Studies have shown that EGFR N-glycosylation critically determines the structural conformation of its ligand-binding ectodomain, which enhances ligand-dependent EGFR activation [35]. Furthermore, N-glycan α2,6 sialylation widely regulates receptor function and signal transduction. Liu et al. reported that EGFR hypersialylation impairs its dimerization and activation and suppresses human lung cancer cell invasiveness [36]. Park et al. reported that increased EGFR N-glycan α2,6 sialylation enhances gefitinib chemoresistance in colon cancer cells [37]. Moreover, it has been shown that the β1 integrins of colon adenocarcinoma cells carry higher levels of α2,6 linked sialic acid and that ST6Gal-I-mediated β1 integrin hypersialylation contributes to colon adenocarcinoma cell adhesion and migration, which indicates that α2,6-sialylation enhances β1 integrin function [38]. Suzuki et al. demonstrated that cleaving cell surface sialic acid via neuraminidase pretreatment promotes human malignant lymphoma adhesion to galectin-3 [39]. It has also been documented that aberrant integrin glycosylation is associated with melanoma progression and that N-glycan β1,6-branched sialylation enhances metastatic melanoma migration [40]. These results indicate that receptor α2,6-sialylation and N-glycosylation greatly regulate malignant cancer cell phenotypes. However, the target proteins of ST6Gal-I in PCa cells still requires further study in our future work.

It is known that Akt phosphorylation induces GSK-3β phosphorylation and inactivation, leading to enhanced Wnt/β-catenin signaling [41]. In our study, ST6Gal-I knockdown inhibited PI3K/Akt/GSK-3β signaling and thus suppressed Wnt/β-catenin signaling. The major components of the Wnt/β-catenin signaling pathway are glycoproteins, such as Wnt proteins and Frizzled-7 receptors. Frizzled-7 receptors are seven-pass transmembrane glycoproteins containing 1 or 2 N-glycosylation sites in their extracellular N-terminal cysteine-rich domain (CRD), which is the Wnt binding domain [42]. Guo et al. also demonstrated that Frizzled receptors are clearly N-glycosylated [43]. Moreover, Wnt family members are found to be widely N-glycosylated. It has been suggested that there are five and two N-glycosylation sites at Wnt 11 and Wnt 3α, respectively, and that deficient N-glycosylation at Wnt 5α and Wnt 3α compromises the secretion of these proteins [44]. Thus, these results indicate that N-glycosylation plays an essential role in Wnt proteins secretion and Wnt/β-catenin signaling. Importantly, these N-glycans serve as potential α2,6-sialylation sites for ST6Gal-I. As mentioned previously, α2,6-linked sialic acid is highly expressed in PCa tissues, and ST6Gal-I knockdown significantly reduces the α2,6-sialylation of almost all sialoglycoproteins. Although the α2,6-sialylation patterns of specific glycoproteins associated with Wnt/β-catenin signaling remain to be identified, it is reasonable to speculate that increased ST6Gal-I expression leads to increased Frizzled and Wnt protein α2,6-sialylation and that Wnt signaling alternations result, at least in part, from decreased Frizzled and Wnt protein α2,6-sialylation.

Metastasis is the leading cause of death in PCa patients, especially bone metastasis. To date, the potential mechanisms underlying PCa bone metastasis remain poorly understood. PC-3 cells are derived from PCa cells that have metastasized to bone, while DU145 cells are derived from PCa cells that have metastasized to the brain. It has been suggested that integrin α2β1, a collagen I receptor and sialylation target, promotes selective PCa bone metastasis [45]. Here, we observed that it's downstream signaling pathway, the PI3K/Akt signaling pathway was much more active in PC-3 cells. Zhu et al. reported that stimulation of PI3K/Akt/NF-kappaB signaling in PC-3 cells upregulates the expression of the steoclastogenesis factors RANK and PTHrP and the osteoblastogenesis factor BMP-2, which may play roles in a variety of bone metastasis-related processes [46–48]. These findings indicate a possible role of ST6Gal-I in PC-3 cell bone metastasis. De Oliveira Barros et al. found that co-culturing DU145 cells with astrocytes significantly increased DU145 cell proliferation and decreased astrocyte proliferation, whereas no significant differences were observed when astrocytes were co-cultured with the lymph node-derived PCa cell line LNCaP [49], indicating that astrocytes can recognize and interact with potential molecules on DU145 cells. Whether glycosylation plays a role in these processes remains to be determined. Thus, it may be worthwhile to determine the role of glycosylation in the differential metastasis of PC-3 and DU145 cells, as well as other tumour cells.

In conclusion, our data indicate that ST6Gal-I expression in PCa tissues is associated with poor survival in patients with PCa. In addition, we found that ST6Gal-I knockdown inhibits the malignant phenotypes of different PCa cell lines through the PI3K/Akt/GSK-3β/β-catenin signaling pathway. Thus, ST6Gal-I overexpression may be a promising target for early PCa diagnosis and treatment.

## MATERIALS AND METHODS

### Clinical tissue samples

Formalin-fixed, paraffin-embedded benign prostate hyperplasia, prostatic intraepithelial neoplasia, and PCa tissues were obtained from 105 patients at the First Affiliated Hospital of Dalian Medical University, and the tumour grades and Gleason scores of these tissues were confirmed by two observers. This study was carried out with the informed consent of all patients and the approval of the ethics committee of Dalian Medical University.

### Immunohistochemistry

After deparaffination in dimethylbenzene overnight and hydration in gradient alcohol, tissue slides were subjected to antigen retrieval in retrieval buffer at 120°C for 5 minutes. Then, the slides were disposed by hydrogen peroxide (3%, 30 minutes) to remove endogenous peroxidase. For ST6Gal-I staining, primary antibodies against ST6Gal-I (Abcam, 1:50) was incubated with the tissue samples for 2 hours at 37°C. For α2,6-linked sialic acid staining, biotin-labelled SNA was diluted as indicated (1:100), and the incubation time was prolonged to 4 hours. After being washed (three washes for 5 minutes each) with PBS, the slides were incubated with biotinylated secondary antibodies for 30 minutes at 37°C. After being washed again, the cells were incubated with HRP-conjugated streptavidin for 30 minutes at 37°C. Then, the cells were treated with DAB (ZSGB-BIO, Beijing, China), which was applied as a developer, before being redyed with haematoxylin and dehydrated with gradient alcohol. Two observers independently evaluated the immunohistochemical staining results according to staining intensities and positive-staining percentages. Positive-staining percentages were classified as lows: negative, 0% (–); weakly positive, 1%–25% (+); moderately positive, 26%–50% (++); and strongly positive, > 50% (+++). Cut-off values were defined as follows: moderately (++) and strongly positive (+++) stained tissue sections were considered to have high protein expression, while negatively (–) and weakly (+) stained sections were considered to have low protein expression in subsequent statistical analysis.

### Cell culture

The human prostate cancer cell lines PC-3 and DU145 were purchased from the Cell Bank of Shanghai Life Science Institution, Chinese Academy of Sciences. PC-3 and DU145 cells were cultured in 1640 medium (Gibco) containing 10% foetal bovine serum (Gibco) in a humidified incubator (Thermo Scientific) at 37°C with 5% CO_2_. For PI3K/Akt/GSK-3β/β-catenin signaling inhibition assay, PCa cells were treated with the PI3K inhibitor wortmannin (Selleck) at 3 μM for 24 h.

### Cell transfection

An RNA interference vector with small hairpin RNA (shRNA) sequences targeting ST6Gal-I and a negative control vector (shNC) were constructed as described previously [50]. Cells in the exponential phase were transfected with a mixture of shRNA or shNC vectors and Lipofectamine™ 2000 (Invitrogen), according to the manufacturer's instructions. Forty-eight hours later, 600 μg/ml G418 (Sigma-Aldrich) was used to screen ST6Gal-I stable silenced cells, and the interference effects were confirmed by RT-PCR, Western blotting and Immunofluorescence assay. Akt shRNA interference vector (Santa Cruz Biotechnology, Inc) and empty control vector (Santa Cruz Biotechnology, Inc) were transiently transfected following their instructions.

### Real-time-PCR

Total RNA was extracted from PC-3 and DU145 cells, shNC and shST6Gal-I voctors transfected PC-3 and DU145 cells by Trizol (Invitrogen) reagent and was reverse-transcribed using a PrimeScript RT Reagent Kit (TaKaRa). Specific primers for glyceraldehyde 3-phosphate dehydrogenase (GAPDH) and ST6Gal-I were purchased from GenePharma. ST6Gal-I mRNA levels were detected by an MX3000P detection system (Stratagene), and the results were analyzed via the 2^--ΔΔCT^ method. GAPDH was used as an internal control.

### Western blot analysis

Cells were lysed using cell lysis buffer (Beyotime) and then quantified using a BCA assay kit (Beyotime). Primary antibodies against p-PI3K (Bioworld), PI3K (Bioworld), ST6Gal-I (Abcam), Akt (Abcam), p-Akt (Abcam), GSK-3β (Affinity biosciences Inc), phosphorylated Ser9 GSK-3β (Affinity biosciences Inc), β-catenin (Santa Cruz Biotechnology, Inc), p-β-catenin (Bioworld), Cyclin D1 (Affinity biosciences Inc), c-Myc (Bioworld), TCF1 (Santa Cruz Biotechnology, Inc), TCF4 (Bioworld), MMP-2 (Santa Cruz Biotechnology, Inc), MMP-7 (Bioworld), MMP-9 (Santa Cruz Biotechnology, Inc), β-actin (Bioworld) and GAPDH (Santa Cruz Biotechnology, Inc) were applied to polyvinylidene fluoride (PVDF) membranes (Pall), and these membranes were subsequently incubated at 4°C for 12 hours at room temperature before being incubated with the appropriate secondary antibody for 2 hours. Protein levels were detected and quantified by an ECL kit (Advansta) and Image Lab software (Bio-Rad).

### Lectin blot analysis

Proteins were extracted using cell lysis buffer (Beyotime). After being quantified with a BCA assay kit (Beyotime), these proteins were boiled with SDS-PAGE protein sample buffer at a ratio of 4:1. Then, a protein mixture containing 35 μg of protein was loaded onto a 10% SDS-polyacrylamide gel, then it was transferred onto an NC membrane. The membrane was blocked in 5% skim milk at 4°C for 12 hours before being incubated with biotin-labelled SNA (which specifically recognizes α2,6-linked sialic acid) at a dilution of 1:2000 at room temperature for 2 hours and peroxidase-labelled streptavidin for 1 hour. Image Lab software (Bio-Rad) was used for detection.

### Immunofluorescence assay

Cells were seeded on coverslips at 30% confluence. After fixation with 4% paraformaldehyde, the cells were incubated with primary antibodies against ST6Gal-I (Abcam, 1:50) and β-catenin (Bioworld, 1:50) for 12 hours at 4°C. After being washed, these cells were incubated with FITC-conjugated goat anti-rabbit second antibody (BD Biosciences). Golgi was localized by GM130 (BD Biosciences, 1:50). After being washed again, the cells were treated with 4′,6-diamidino-2-phenylindole (DAPI) and imaged under a confocal laser scanning microscope (BD Biosciences).

### Flow cytometry analysis

Cells were harvested and fixed with 75% alcohol. Then, the cells were blocked with 1% bovine serum albumin (BSA) and incubated with 2 μg/ml FITC-conjugated SNA lectin on ice for 30 minutes. After being washed three times with PBS, the cells were analyzed with a flow cytometer (BD Biosciences).

### CCK8 and cell colony formation assay

Approximately 2000 cells were seeded into a 96- well plate in triplicate, and 10 μl of CCK8 reagent in 100 μl of 1640 medium with 10% FBS was added into each well for 4 hours of incubation at 37°C with 5% CO_2_. A TECAN microplate reader (Tecan, Mechelen, Belgium) was used to measure the OD values at 450 nm. For cell colony formation assay, 5000 cells were seeded in six-well plates and incubated for 14 days at 37°C with 5% CO_2_ in 1640 medium with 10% FBS. After being stained with 0.1% crystal violet and washed, the plates were photographed. We randomly selected ten different fields in each six-well plate at 400 × magnification and counted the numbers of cell clusters comprising more than 50 cells.

### Cell scratch assay

Four lines were evenly marked on the back of each well of each six-well plate to ensure that the same areas were photographed at every time point. Then, approximately 2.5 × 10^6^ cells were transferred to each well. After these cells reached confluence, a 200 μl sterile pipette tip was used to make a gap in the monolayer. Then, the cells were washed with 1640 medium without FBS and photographed immediately with a microscope at 100 × magnification. After the cells were cultured for 16 hours in 1640 medium without FBS, the same areas were photographed again.

### Transwell migration and invasion assay

About 5 × 10^4^ cells in 150 μl of 1640 medium without FBS were added to the upper compartment of a 24-well transwell chamber (pore size 8.0 μm, diameter 6.5 mm, Costar, USA), and 350 μl of 1640 medium with 20% FBS was added to the lower compartment. After incubating for 9.5 hours with 5% CO_2_ at 37°C, the upper chamber was washed three times with PBS, and the cells were subsequently stained and fixed with 0.1% crystal violet diluted with methanol for 2 hours. After being washed three times with PBS and dried, the cells were photographed with a microscope at 200× magnification. Finally, Image-Pro Plus 6.0 software was used to count the numbers of cells in four randomly chosen areas. For invasion assay, all the procedures were the same as those described above; however, the 24-well transwell chambers were coated with 40 μl of Matrigel (at a dilution of 1 to 8) on the upward-facing side of the polycarbonate membrane, 1.5 × 10^5^ cells were added to upper chamber, and the culture time was prolonged to 48 hours.

### Statistical analysis

Statistical analysis was performed using SPSS 13.0 software. Continuous variables were expressed as the mean ± SD. The relationship between ST6Gal-I expression and clinicopathological characteristics was analyzed by Pearson's *χ*^2^ and Fisher's exact tests. The correlation between ST6Gal-I expression in PCa tissues and clinicopathological characteristics was determined by Spearman correlation analysis. Overall survival (OS) was defined as the time from surgery to death or the last follow-up. Progression-free survival (PFS) was defined as the time from treatment to recurrence or progression (diagnosed via imaging or clinical assessments). Kaplan–Meier curves were plotted to calculate the effects of ST6Gal-I expression on PFS and OS, and survival curves were compared using a log-rank test. Cox regression (proportional hazard model) was performed for multivariate analysis of prognostic predictors. Differences between groups were compared using Student's *t* test, and differences among three or four groups were analyzed using one-way ANOVA. Two-sided *P* values < 0.05 were considered statistically significant.

## SUPPLEMENTARY MATERIALS TABLES


