# Supplementary material for: ST6Gal-I overexpression facilitates prostate cancer progression via the PI3K/Akt/GSK-3β/β-catenin signaling pathway
Source: Oncotarget. 2016 Aug 30;7(40):65374–88. doi: 10.18632/oncotarget.11699 (PMC5323162; doi:10.18632/oncotarget.11699)
Supplement: Supplementary file 1 [file oncotarget-07-65374-s001.pdf]

ST6Gal-I overexpression facilitates prostate cancer progression via the PI3K/Akt/GSK-3β/β-catenin signaling pathway

Supplementary Materials

Supplementary Table S1: Prognostic value of ST6Gal-I expression for overall survival in univariate and multivariate analyses by Cox regression

Overall survival	Univariate analysis		Multivariate analysis	
	HR (95% CI)	*P* Value	HR (95% CI)	*P* Value
Age	1.332 (0.689–2.575)	0.394	-	-
Pretreatment PSA	2.776 (1.422–5.417)	0.003	1.541 (0.732–3.245)	0.255
Gleason score	0.369 (0.216–0.633)	< 0.001	0.634 (0.358–1.124)	0.119
Pathologic stage	2.669 (1.655–4.305)	< 0.001	2.119 (1.063–4.226)	**0.033**
Lymph node metastases	3.053 (1.492–6.247)	0.002	0.828 (0.307–2.232)	0.710
ST6Gal-I expression	4.317 (2.038–9.146)	< 0.001	3.603 (1.548–8.387)	**0.003**
Seminal vesicle involvement	3.152 (1.563–6.357)	0.001	1.469 (0.691–3.123)	0.318
Perineural invasion	1.068 (0.524–2.177)	0.856	-	-

Supplementary Table S2: Prognostic value of ST6Gal-I expression for progression-free survival in univariate and multivariate analyses by Cox regression

Progression-free survival	Univariate analysis		Multivariate analysis	
	HR (95 % CI)	*P* Value	HR (95% CI)	*P* Value
Age	1.384 (0.654–2.929)	0.395	-	-
Pretreatment PSA	2.613 (1.224–5.577)	0.013	1.604 (0.690–3.730)	0.272
Gleason score	0.376 (0.208–0.680)	0.001	0.598 (0.312–1.144)	0.120
Pathologic stage	2.097 (1.222–3.598)	0.007	1.634 (0.899–2.971)	0.107
Lymph node metastases	1.677 (0.674–4.170)	0.266		
ST6Gal-I expression	2.873 (1.305–6.325)	0.009	2.389 (1.030–5.542)	**0.042**
Seminal vesicle involvement	2.118 (0.969–4.630)	0.060		
Perineural invasion	1.071 (0.484–2.372)	0.865	-	-
